# Human macrophages infected with Egyptian Rousette bat-isolated Marburg virus display inter-individual susceptibility and antiviral responsiveness

**DOI:** 10.1038/s44298-024-00027-3

**Published:** 2024-05-02

**Authors:** Ivet A. Yordanova, Angelika Lander, Annette Wahlbrink, Jonathan S. Towner, César G. Albariño, Lay Teng Ang, Joseph B. Prescott

**Affiliations:** 1 Center for Biological Threats and Special Pathogens, Robert Koch Institute, 13353 Berlin, Germany; 2 Viral Special Pathogens Branch, Centers for Disease Control and Prevention, Atlanta, GA 30329 USA; 3 Stanford Institute for Stem Cell Biology & Regenerative Medicine, Stanford University, Stanford, CA 94305 USA

**Keywords:** Monocytes and macrophages, Marburg virus, Viral host response, Virus-host interactions

## Abstract

Marburg virus (MARV) is a highly pathogenic filovirus and a causative agent of sporadic zoonotic viral hemorrhagic fever outbreaks with high case fatality rates. In humans, filoviruses like MARV and Zaire Ebola virus (EBOV) target, among others, innate immune cells like dendritic cells and macrophages (MΦs). Filovirus-infected dendritic cells display impaired maturation and antigen presentation, while MΦs become hyper-activated and secrete proinflammatory cytokines and chemokines. Our current understanding of human macrophage responses to MARV remains limited. Here, we used human monocyte-derived macrophages (moMΦs) to address how their phenotype, transcriptional profile, and protein expression change upon an in vitro infection with a bat isolate of MARV. Confirming its tropism for macrophages, we show that MARV induces notable shifts in their transcription distinct from responses induced by lipopolysaccharide (LPS), marked by upregulated gene expression of several chemokines, type I interferons, and IFN-stimulated genes. MARV infection also elicited pronounced inter-individually different transcriptional programs in moMΦs, the induction of Wnt signaling-associated genes, and the downregulation of multiple biological processes and molecular pathways.

## Introduction

Marburg virus (MARV) is a highly pathogenic zoonotic filovirus and an etiologic agent of sporadic outbreaks of viral hemorrhagic fever with high case fatality rates^1–3^. Similar to Zaire Ebola virus (EBOV), early MARV infection typically results in general flu-like symptoms in humans, rapidly progressing to a severe and often fatal Marburg virus disease (MVD). This is often accompanied by an early and substantial loss of lymphocytes, unchecked secretion of proinflammatory cytokines and chemokines, suppressed type I interferon (IFN) responses, and delayed antibody production^3,4^.

Among the key initial targets of filoviruses are host innate immune cells of the myeloid compartment, such as dendritic cells (DCs) and macrophages (MΦ)^5^. Upon infection by EBOV, DCs exhibit arrested maturation, inadequate antigen presentation, and deficient IFN responses. In contrast, infected MΦs display uncontrolled activation and release high concentrations of tissue-damaging proinflammatory cytokines and chemokines^3,6–8^. Similar findings have been confirmed in murine models^9,10^, humanized mice^11^ and non-human primates^12,13^. Host DCs and MΦs, therefore, not only enable initial filovirus replication and dissemination, but also contribute to pathogenesis via inappropriate antiviral immune responses in the early phase of infection^8^.

Despite the overall similar symptomatology and progression of Ebola virus disease (EVD) and MVD, at the molecular level, EBOV and MARV only share a 35% nucleotide sequence identity and exhibit several notable differences in their viral protein structure and functions^14–16^. Studies of DC and MΦ responses to filoviruses have almost exclusively focused on EBOV, while our understanding of MARV interactions with human innate immune cells and how these might differ from responses to EBOV remains incomplete. Past work has shown that MARV efficiently infects and replicates in human monocyte-derived macrophages (moMΦs) and elicits TNF secretion in vitro, while MARV and EBOV-infected humanized mice display differential MΦ expansion, maturation, and activation^8,17^. Together, these findings highlight that these two filoviruses exhibit different effects on host myeloid cells. Hence, MARV-induced host responses cannot be simply extrapolated from prior EBOV studies. Furthermore, how human innate immune cells respond to a bat isolate of MARV remains unknown^18–20^. Herein, we aimed to characterize the early human MΦ responses to an in vitro MARV infection using a bat isolate of MARV.

## Results

### Human moMΦs are permissive to MARV infection

**Fig. 1:**
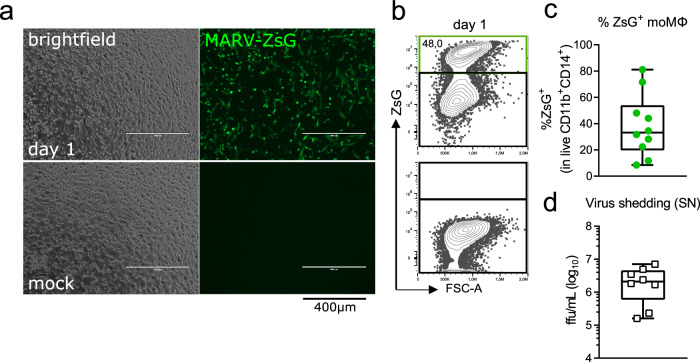
MARV replication and progeny production in human moMΦs 1 day following MARV-ZsG infection. **a** ZsG expression in MARV-infected versus mock-infected human moMΦs, as observed under a fluorescent microscope. **b** Example FACS plots and **c** percentages of ZsG^+^ cells within live CD11b^+^CD14^+^ moMΦs in individual donors, quantified using flow cytometry. **d** Virus production in cell culture supernatants of MARV-infected moMΦs, quantified using a focus assay. The data are pooled from two independent experiments with *n* = 3–5 donors per experiment.

Upon inoculation with recombinant bat MARV371 (isolate Uganda 200704852) expressing fluorescent ZsGreen protein (MARV-ZsG) at a MOI of 2, measured using Vero E6 cells, we show that in vitro-differentiated moMΦs are permissive to infection, with an average 50% of cells expressing ZsG protein within 1 day of infection (Fig. 1a). To better quantify the proportions of virus-infected moMΦs at the individual donor level, we performed flow cytometry of MARV-ZsG-infected cells, including mock-infected samples as a reference for the ZsG signal (Fig. 1b). Despite MARV-ZsG successfully infecting moMΦs derived from all donors, the frequencies of ZsG^+^ cells within the CD11^+^CD14^+^ population varied from 10-80%, revealing drastic inter-individual differences in overall moMΦ permissiveness to MARV (Fig. 1c). In line with this finding, virus progeny production in cell culture supernatants also highly differed between individual donors (Fig. 1d).

### Virus-infected moMΦs upregulate their surface expression of the scavenger receptor CD163

**Fig. 2:**
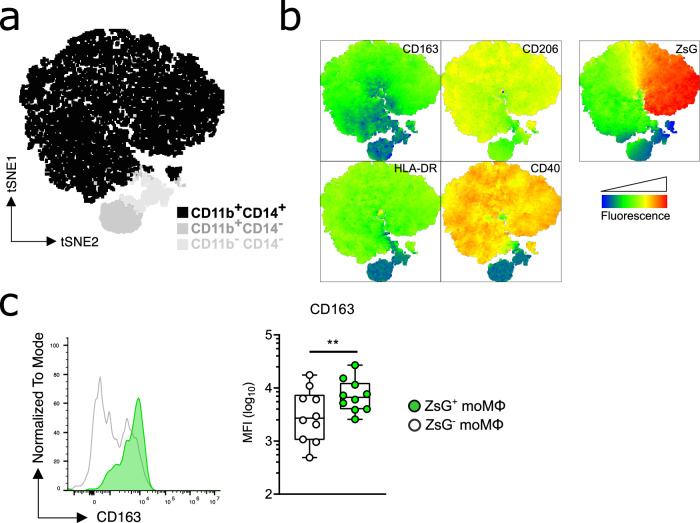
Cell surface marker expression in moMΦs 1-day post-infection with MARV-ZsG. **a** Dimensionality reduction analysis (tSNE) of the cell populations found in in vitro-differentiated human moMΦ cultures. **b** Overlay tSNE plots illustrating the expression patterns of macrophage surface markers CD163, CD206, HLA-DR, and CD40, as well as of ZsG, within MARV-infected moMΦ cultures. **c** Example histogram plot and median fluorescence intensity (MFI) of the surface expression levels of CD163 in ZsG^+^ and ZsG^-^ moMΦs. The results in **c** are pooled from two independent experiments with *n* = 3–5 donors per experiment. Statistical analysis was done using a Wilcoxon Signed Rank test. **p < 0.01.

To further characterize the cell subset composition of our in vitro-differentiated moMΦs and to explore whether MARV-ZsG affects their expression of various markers, we assessed moMΦ surface marker expression by flow cytometry (Supplementary Fig. 1c, d). Dimensionality reduction analysis of live singlets in our moMΦ cultures demonstrated a predominance of CD11b^+^CD14^+^ cells, confirming the consistency of our moMΦ differentiation method (Fig. 2a). The expression of typical moMΦ markers such as the scavenger receptor and activation marker CD163, the alternatively activated M2 macrophage marker CD206 and antigen presentation markers such as HLA-DR and CD40 were almost entirely expressed within the CD11b^+^CD14^+^ population (Fig. 2b). MARV-ZsG exclusively infected CD163^+^CD206^+^MHC-II^+^CD40^+^ cells, confirming its selective tropism for moMΦs (Fig. 2b).

Elevated plasma levels of the secreted form of CD163 (sCD163) have previously been associated with severe and fatal EBOV infections in humans^21^. To check whether MARV-ZsG induces a similar CD163 response in human moMΦs, we quantified the median fluorescence intensity (MFI) of surface CD163 expressed on virus-infected (ZsG^+^) and bystander (ZsG^-^) cells by flow cytometry and assessed sCD163 concentrations in cell culture supernatants. We found that MARV-ZsG-infected cells expressed significantly higher levels of surface CD163 compared to uninfected bystander cells at 1 day post-infection (Fig. 2c), albeit in the absence of increased sCD163 in cell culture supernatants (Supplementary Fig. S2b).

### MARV induces prominent shifts in the transcriptional profile of human moMΦs

**Fig. 3:**
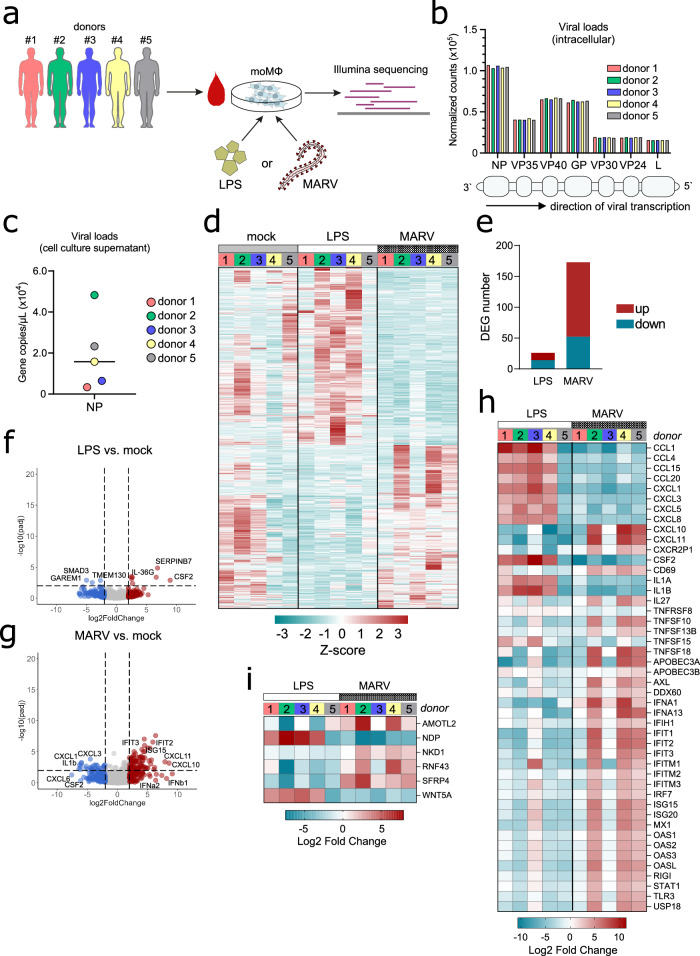
Differential gene expression in LPS-treated and MARV-infected human moMΦs. **a** moMΦs were differentiated from five individual healthy blood donors. Cells from each donor were either mock-infected, LPS-treated, or MARV-infected and were harvested after 1 day of treatment or infection for bulk RNAseq analysis. **b** Normalized counts of all 7 MARV genes in individual donors, measured using RNA sequencing. **c** Viral production in cell culture supernatants, quantified using qRT-PCR detection of MARV NP and shown as gene copies per µL cell culture supernatant. **d** Global gene expression profile of mock-infected, LPS-treated, and MARV-infected moMΦs from individual donors. **e** Total numbers of significantly upregulated and downregulated DEGs in moMΦs following in vitro LPS or MARV challenge (filtered for treatment group-level *p*-adjusted values < 0.05). Volcano plots illustrating significant DEGs across all five donors in (**f**) LPS-treated and (**g**) MARV-infected cells, compared with mock-infected controls. Genes upregulated twofold or higher are highlighted in red, while genes downregulated twofold or lower are highlighted in blue. **h** Heatmap illustrating immune-related DEGs in LPS-treated and MARV-infected moMΦs. **i** Heatmap illustrating Wnt signaling-related DEGs in LPS-treated and MARV-infected moMΦs. The heatmaps in **h** and **i** are shown as log_2_ fold change against mock-infected controls.

To characterize the transcriptional profile of human MΦ responses to a bat isolate of MARV, in vitro differentiated moMΦs were infected with wild-type MARV371 (MARV) at a MOI of 2. As a positive control to assess general MΦ activation, cells were exposed to the common immune agonist lipopolysaccharide (LPS), while mock-infected cells served as negative controls. We sequenced bulk RNA from moMΦs derived from 5 individual healthy blood donors (Fig. 3a). Both MARV and MARV-ZsG replication in primary human MΦ has previously been compared, demonstrating similar intracellular replication and growth kinetics up to 3 days post-infection^22^.

To assess the cell subset composition of our cell cultures, the normalized log_2_ gene counts obtained from RNA sequencing were analyzed using the online tool CIBERSORTx. For all 5 sequenced donors, over 90% of cells were classed as macrophages based on their gene expression profile under mock conditions (Supplementary Fig. 3). Analysis of viral replication in MARV-infected moMΦs revealed overall similar gene copy numbers of all 7 MARV genes in individual donor samples, indicative of comparable intracellular viral replication (Fig. 3b). In contrast, quantification of viable virus in cell culture supernatants revealed notable inter-individual differences in viral production, congruent with our observations of susceptibility differences in MARV-ZsG-infected cells (Fig. 3c).

Overall, RNAseq analysis revealed significantly disparate transcriptional profiles are induced between LPS-treated and MARV-infected moMΦs (Fig. 3d). Noteworthy inter-individual differences in global gene expression profiles were also evident, with individual donors displaying variable gene expression profiles for each condition. Specifically, while moMΦs from donor 5 displayed negligible shifts in gene transcription in response to LPS, cells from this donor had a pronounced response to MARV. In contrast, cells from donors 1 and 3 displayed a contrastingly weak response to MARV, but dramatic changes in gene expression following LPS treatment (Fig. 3d).

MARV infection induced the downregulation of 52 and the upregulation of 121 differentially expressed genes (DEGs), notably fewer than LPS (Fig. 3e–g). While LPS treatment upregulated genes for several proinflammatory cytokines and CCL-family and CXCL-family chemokines, *CSF2*, *IL1A*, and *IL1B* in 4 out of 5 donors, MARV infection induced a markedly different transcriptional profile in moMΦs (Fig. 3h). Examining immune response-associated DEGs at the individual donor level, two distinct groups of virus non-responders and responders were apparent in our data. Donors 1 and 3 displayed an overall unimpaired cellular response to LPS treatment, but no significant upregulation of immune-related genes was observed in response to MARV, thus marking these donors as MARV non-responders. In contrast, donors 2, 4, and 5 (MARV responders) displayed distinct and consistent upregulation of a diverse set of genes, including chemokines *CXCL10* and *CXCL11*, the activation marker *CD69*, the immunomodulatory cytokines *IL27* and *TNFSF18*, and several Type I IFN response genes, including *IFNA1* and *IFNA13*, *IRF7*, *ISG15*, and *ISG20* and *OAS1*, *OAS2*, and *OAS3* among others (Fig. 3h).

### MARV-infected human moMΦs show differential expression of Wnt signaling-associated genes

The Wnt signaling pathway is a highly-conserved complex network of intracellular signaling cascades, dictating diverse physiological processes associated with cell differentiation, migration, and survival in various tissues^23^. Here, we found that LPS and MARV induce different gene expression profiles of several Wnt signaling-associated genes. MARV infection triggered significant upregulation of *AMOTL2*, *NKD1*, *RNF43*, and *SFRP4*, while LPS only induced moderately elevated gene expression of *NDP* and *WNT5A*, both of which were downregulated in response to MARV (Fig. 3i).

### MARV non-responders and responders have distinct baseline transcriptional profiles

**Fig. 4:**
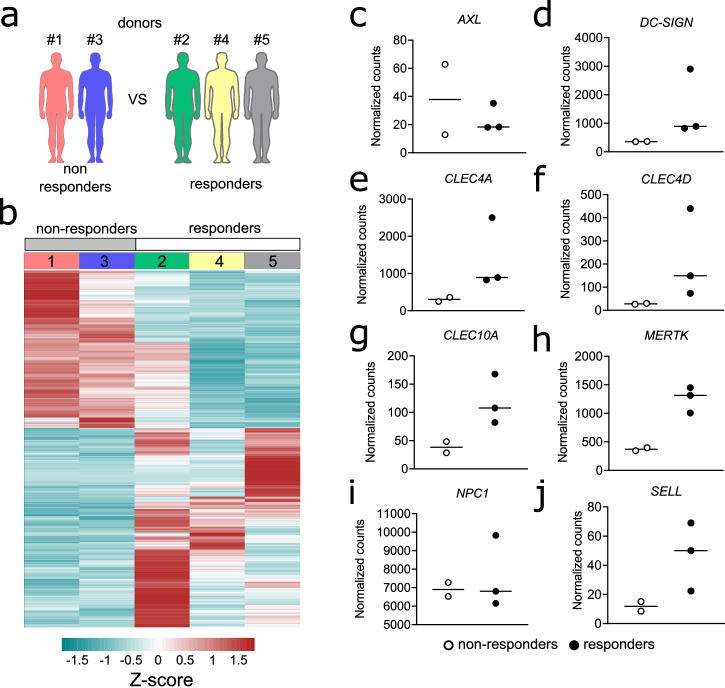
Baseline global gene expression and cell surface receptor expression in virus non-responders versus responders. **a** RNAseq analysis set-up, grouping and comparing MARV non-responder donors 1 and 3 versus responder donors 2, 4, and 5 at baseline (mock-infected control samples). **b** Global gene expression in individual non-responder versus responder donors. **c**–**j** Normalized gene counts of genes encoding cell surface receptors associated with filovirus attachment and entry.

Considering the observed inter-individual differences between donors in response to MARV, we assessed whether these virus non-responder and responder moMΦs have differences in their baseline transcriptional profiles, leading to their differential response to MARV. We re-analyzed their global transcriptional profile using mock-infected moMΦs as a reference, comparing directly the two MARV non-responders (donors 1 and 3) and the three MARV responders (donors 2, 4, and 5) (Fig. 4a). We observed two distinct transcriptional profiles, with non-responders showing a largely consistent transcriptional profile. Similarly, despite some observable differences, the overall baseline (pre-infection) transcriptional profile of donors 2, 4, and 5 was notably distinct from that of non-responders (Fig. 4b, Table S1).

We then investigated whether, at baseline, MARV non-responders and responders express variable levels of receptors associated with filovirus attachment or entry. We examined gene copy numbers of *AXL, DC-SIGN*, and *NPC1*, as well as several C-type lectins (Fig. 4c–j). We found greater expression of several of these receptors in MARV responders, compared to non-responders. Namely, *DC-SIGN, CLEC4A*, *CLEC4D*, *CLEC10A*, *MERTK*, and *SELL* all displayed higher gene copy numbers in the three responders, compared with the two non-responders, indicative of baseline differences in susceptibility of certain individuals to filovirus infection at the cellular level in vitro (Fig. 4d–h, j).

### Human moMΦs are biologically dysregulated following MARV infection

**Fig. 5:**
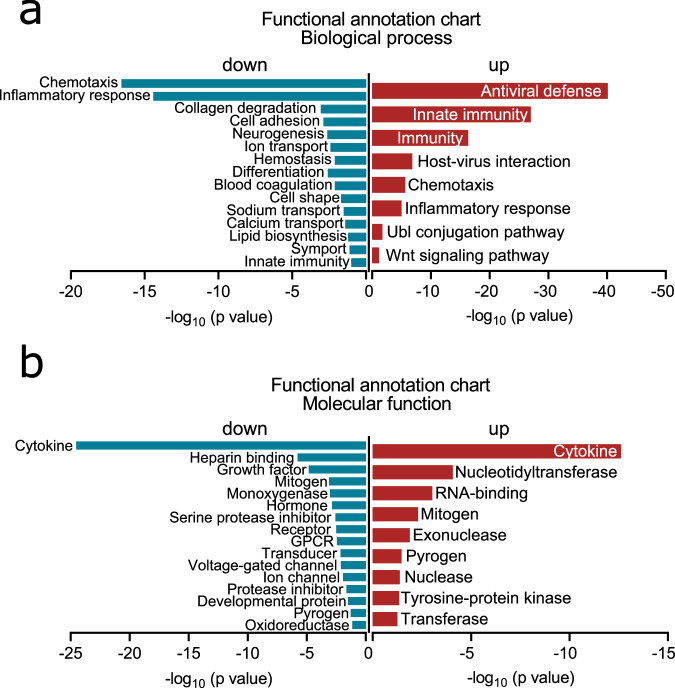
Functional annotation analysis of DEGs in MARV-infected moMΦs (differentially expressed against mock-infected controls). **a** Biological process and **b** Molecular function gene ontology (GO) terms, enriched among DEGs in MARV-infected moMΦs. GO terms enriched within genes upregulated twofold or higher are shown in red, while genes downregulated twofold or lower are highlighted in blue. Functional annotation analysis was performed using DAVID.

To further characterize the shifts in the transcriptional profile of MARV-infected moMΦs, we performed gene ontology analysis of DEGs. For this, functional annotation of DEGs across biological process and molecular function gene ontology (GO) terms was performed using DAVID^24,25^. While upregulated genes in response to MARV were enriched across 8 GO terms, including *antiviral defense*, *innate immunity* and *host–virus interaction*, downregulated genes belonged to almost twice as many categories (Fig. 5a). GO terms associated with significantly downregulated genes included *chemotaxis*, *inflammatory response*, *cell adhesion*, *ion transport*, *differentiation* and *cell shape*, demonstrating that alongside eliciting antiviral immune responses, MARV infection simultaneously disrupts various cellular processes necessary for efficient immune cell responses (Fig. 5a).

Functional annotation of GO terms across molecular functions revealed similar findings, with upregulated genes being enriched across 9 GO terms, while downregulated genes were enriched across 16 GO terms. Molecular functions such as *cytokine*, *heparin-binding, growth factor*, and *voltage-gated channel* were all enriched among significantly downregulated genes in MARV-infected samples. Together, these findings highlight that despite the induction of chemokine and type I IFN responses, moMΦs experience parallel suppression of various homeostatic molecular functions following MARV infection (Fig. 5b).

### Responses to MARV at the protein level correspond with observed shifts in moMΦ transcription

**Fig. 6:**
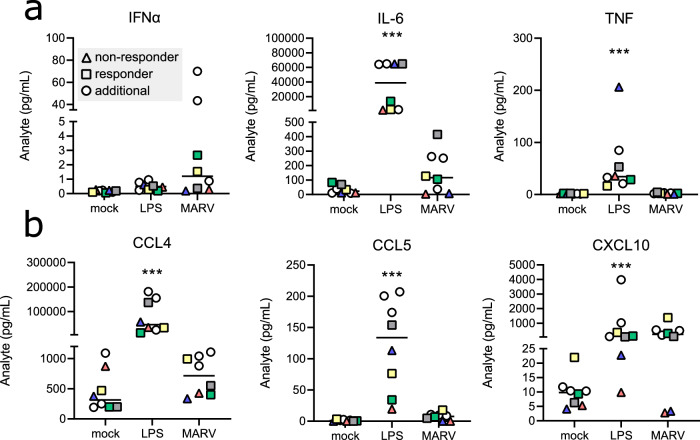
Cytokine and chemokine expression in human moMΦs. Protein levels of **a** proinflammatory cytokines IFNα, IL-6, and TNF and **b** chemokines CCL4, CCL5, and CXCL10 in cell culture supernatants of mock-infected, LPS-treated and MARV-infected moMΦs 1 day post-treatment or infection, measured using a 34-plex human Luminex assay. Donors defined as MARV non-responders are marked as triangles, while responder donors are marked in squares. Supernatants from three additional donors (white circles) were included in the Luminex assay. The data are pooled from 2 independent experiments with =3–5 donors per experiment. Statistical analysis was performed using a Kruskall–Wallis multiple comparisons test. ***p < 0.001.

Many proinflammatory cytokines and chemokines undergo post-transcriptional or post-translational modifications, and RNAseq data alone may not be sufficient to profile certain innate immune responses. Therefore, we assessed moMΦ cytokine and chemokine responses to MARV at the protein level. For this, we analyzed cell culture supernatants from mock-infected, LPS-treated, and MARV-infected cells using a 34-plex Luminex assay (Table S2). Compared with LPS treatment, we observed an overall limited protein secretion from MARV-infected cells. MARV induced low-level secretion of IFNα and the proinflammatory cytokine IL-6, while significant TNF production was only detected in response to LPS exposure (Fig. 6a). Similarly, secretion of CCL4 and CCL5 was significantly increased in LPS-treated, but not MARV-infected moMΦs, in line with the gene expression kinetics for both chemokines. However, we observed a marked increase in CXCL10 production in virus-infected cells, comparable to that detected in LPS-treated moMΦs and congruent with the transcriptional changes (Fig. 6b). Considering the non-responder and responder genotypes outlined herein, we also observed notable differences in protein expression between individual donors. The two non-responder donors secreted markedly lower amounts of CXCL10 both in response to LPS stimulation and MARV infection (Fig. 6b). In contrast, protein secretion of the remaining cytokines and chemokines examined showed no notable distinction between non-responders and responders.

## Discussion

Little is known regarding human innate immune responses to MARV, with the few studies published to date having exclusively examined human virus isolates. Considering the zoonotic origin of MARV, harbored by the Egyptian Rousette bat, and the high sequence similarity of the MARV371 bat isolates to MARV-Angola isolates^26^, the aim of our current study was to characterize how human innate immune cells respond to MARV371 (MARV) upon potential spillover from a bat reservoir. For this, we used moMΦs to profile MARV tropism and replication efficiency, virus-induced shifts in cell phenotype, gene expression, and protein secretion and how those might differ at the inter-individual level.

We show that human moMΦs are susceptible to an in vitro infection with a bat isolate of MARV, with notable inter-individual differences in infection rates and viral production. MARV-infected moMΦs significantly increased their surface expression of CD163, compared with bystander cells. CD163 is a scavenger receptor for hemoglobin–haptoglobin (HbHp) complexes, an innate immune sensor for bacteria^27^ and a viral attachment and internalization receptor^28^. Moreover, elevated levels of secreted CD163 (sCD163) have been associated with both EVD and hantavirus-induced hemorrhagic fever with renal syndrome^29,30^. Here, we found that compared with bystander cells, MARV-infected moMΦs undergo a pronounced increase in CD163 surface expression, albeit in the absence of elevated sCD163 levels. This suggests that in vivo filovirus-induced secretion of sCD163 from activated macrophages could be reliant on additional signaling that is absent in an in vitro infection setting.

EBOV and MARV are etiologic agents of viral hemorrhagic fevers with an overall similar clinical picture, rapid progression, and an often fatal outcome. Macrophage tropism is a key characteristic of both filoviruses. Prior work has shown that EBOV-infected human moMΦs secrete TNF and CXCL10, paralleled by a lack of Type I IFN release^31,32^. Starting at 1-day post-infection, EBOV induces defined shifts in the transcriptional profile of human moMΦs, including the upregulated gene expression of various Type I IFN genes, *OASL, ISG15*, and *ISG20, IFIT1, IFIT2* and *IFIT3*, and chemokines like *CCL5* and *CXCL10*, all of which were also found as significantly upregulated in our own findings for MARV. However, EBOV was shown to significantly induce the upregulation of a more diverse set of *IFNA* and *IFNB* genes and a larger number of chemokines, including *CCL3, CCL4, CCL19, CCL20*, *CXCL6*, and *CXCL8* at 1 day post-infection^32^. Herein, MARV induced the significant upregulation of only two IFNA genes (*IFNA1* and *IFNA13*) and two chemokines (*CXCL10* and *CXCL11*), highlighting a more muted moMΦ gene expression profile in response to MARV compared to findings for EBOV^32^. Whether this differential response relates to a general difference in moMΦ responses to EBOV and MARV or signals to a muted human innate immune response to a bat isolate of MARV merits further investigation.

Shared genetic variants across individuals are important drivers of host cellular responses to pathogens, and the contribution of host genetics to inter-individual differences in innate immune responses is increasingly being recognized^33^. For filoviruses, clear inter-individual differences in proinflammatory cytokine, chemokine, and IFN responses have previously been shown for EBOV-infected moMΦs^32,34^ Herein, we show not only that human moMΦs display variable permissiveness to MARV infection in vitro, but also that MARV-infected moMΦs from our small donor cohort display two distinct transcriptional profiles. Among the 5 sequenced donors, two were clear MARV non-responders, showing no significant shifts in immune-related gene expression following MARV infection. Cells from the remaining 3 donors (MARV responders) displayed a contrasting response to MARV. This was characterized by the significant upregulation of the expression of various activation markers, chemokines, type I IFN responses, and ISGs, expanding on previous microarray-based studies of human macrophage responses to MARV^35^. Moreover, these inter-individual differences in moMΦs were inconsistent between LPS treatment and MARV infection, demonstrating that the observed innate immune responses to MARV are highly specific, not a function of generalized responsiveness differences to immune-stimulating ligands, and can significantly differ among individuals. Consistent with the gene expression profile of these donors, qRT-PCR quantification of MARV NP in cell culture supernatants revealed an almost 6-fold difference in viral production in moMΦs between the two non-responders (donors 1 and 3) and the three responders (donors 2, 4, and 5, Fig. 3c). Similar responses at the gene expression level to those observed in our MARV responder cohort have been measured within the first 3 days of a MARV infection in rhesus and cynomolgus macaques, confirming the existence of common MARV-induced innate immune responses, shared among humans and non-human primates^36,37^.

Host antiviral immune responses are governed by complex, multi-step molecular interactions and signaling cascades. Differences in baseline genetic parameters such as gene dose effects affecting pathogen-associated molecular pattern (PAMPs) recognition by TLRs, HLA haplotypes, or single nucleotide polymorphisms (SNPs) in key viral entry receptors can contribute to pronounced inter-individual differences in responses to a subsequent viral infection^38,39^. Herein we provide evidence of differences in the baseline gene expression profiles of moMΦs derived from 5 healthy individuals, affecting in vitro infection with MARV. We found that compared with the two MARV non-responders, under mock infection conditions, the three responder donors displayed significantly upregulated expression of a number of *HLA* genes, as well as *TLR2*, suggestive of potential baseline differences in the ability of moMΦs from different individuals to recognize and present foreign antigen and hence to induce downstream antiviral immune responses (Table S1).

Filoviruses typically enter host cells through a complex sequence of steps, which have not been fully elucidated. Several host cell receptors are known to facilitate filovirus entry, such as various C-type lectins (CLECs) that bind viral glycoprotein (GP), members of the TAM family of receptors such as Axl, Mer and Tyro3, Niemann-Pick disease, type C1 (NPC1), dendritic cell-specific ICAM-3-grabbing non-integrin (DC-SIGN) and macrophage galactose-type lectin (MGL)^8,40^. While their expression can vary considerably between cell subsets and tissue compartments, less is known about inter-individual variability in filovirus receptor expression and how that might influence individual susceptibility or responses to infection. Herein, we also found pronounced differences in gene expression of several of these receptors between MARV responder and non-responder donors prior to infection. We observed greater gene expression of *DC-SIGN*, three CLEC-family lectins, *SELL* (encoding the cell adhesion receptor CD62-L) as well as *MERTK* (encoding a TAM family receptor) in all three MARV responders, compared with the non-responder group. This lower baseline expression of filovirus entry-associated genes corresponds with both the lower virus progeny and the immunologically silent state of non-responder moMΦs.

Wnt signaling pathways are highly conserved intracellular cascades governing diverse functions, ranging from body axis patterning and cell fate specification to cell proliferation and cell migration^22^. Various studies have previously demonstrated a connection between Wnt signaling and viral infections, showing either virus-driven overt stimulation or inhibition of Wnt signaling^41^. Considering the strong deregulation of innate immune cell maturation, proliferation, and antiviral responses described for filoviruses like EBOV or MARV^5^, the presence of filovirus-induced shifts in Wnt signaling is, therefore, conceivable but remains unaddressed to date. Herein, we describe for the first time the differential expression of several genes associated with Wnt signaling in MARV-infected human moMΦs. Among these was *AMOTL2*, which encodes an angiomotin membrane-associated scaffold protein involved in endothelial cell elongation, migration, junction formation, and apical polarity^42–44^. The human *AMOTL2* gene encodes two protein isoforms, p100 and p60, the latter shown to induce loss of cell polarity and broad disruption of tissue architecture in human breast and colon cancers^43^. Considering that endothelial cell damage, disrupted vascular permeability, and cell dysfunction are factors associated with filovirus hemorrhagic fevers, a potential role of MARV-induced *AMOTL2* overexpression for the induction of endothelial cell damage is conceivable. Cell culture supernatants collected from MARV-infected human monocyte/macrophage cultures have been shown to increase endothelial cell permeability, indicating that MARV-infected macrophages secrete factors influencing endothelial cell permeability^16^. Whether AMOTL2 is potentially among these factors merits further investigation.

Our study also provides a new perspective on the various cellular and molecular processes targeted and disrupted by MARV. GO terms enriched among downregulated DEGs in MARV-infected moMΦs included *chemotaxis, cell adhesion*, and *differentiation*, as well as various GO terms associated with ion transport across cell membranes. This suggests that MARV-infected moMΦs potentially undergo altered chemotaxis and broad disruptions in cell metabolism. Fitting with the gene expression data and with the known antagonistic properties of filoviral proteins, we observed only weak IFNα protein secretion in MARV-infected samples^35^. In contrast, increased CXCL10 protein levels corroborated the gene expression data and highlighted CXCL10 as the major chemokine involved in moMΦ in vitro responses to MARV.

In summary, we report for the first time how the cell phenotype, transcriptional profile, and protein secretion of human moMΦs change within the first day of infection with the original bat isolate of MARV. We show that despite known differences between EBOV and MARV sequence identity and virion structure, MARV induces overall similar innate immune responses to those previously reported for MARV in non-human primates and for EBOV in humans. On the other hand, we also demonstrate the presence of marked inter-individual differences in moMΦs permissiveness, virus shedding, and transcriptional profiles between individual donors in response to MARV. Finally, we highlight that the non-responder and responder donors in our study display notably different transcriptomes at baseline, indicating that host genetics might play a significant role in predisposing individuals to variable susceptibility and immune responses to MARV.

## Methods

### Blood sampling, PBMC, and CD14^+^ monocyte isolation

Approximately 10 mL of fresh blood was collected in EDTA blood collection tubes (Sarstedt) from healthy human donors through the blood donation service of the Center for Transfusion Medicine and Cell Therapy (ZTB) Berlin GmbH, part of Charité Universitätsmedizin Berlin. Samples were collected under ethics approval from the Ethics Committee of the Charité Universitätsmedizin Berlin (approval EA2/227/22 granted to IAY). Written informed consent was obtained from all donors.

Within 2 h of blood collection, peripheral blood mononuclear cells (PBMCs) were isolated using Lymphoprep^TM^ density gradient separation following the manufacturer’s instructions (StemCell Technologies). Freshly isolated PBMCs were counted and labeled with human anti-CD14 MicroBeads (Miltenyi Biotec), followed by magnetic bead separation and enrichment for positive selection of CD14^+^ monocytes (Supplementary Fig. 1a, b). For magnetic labeling, washed PBMCs were resuspended in 80 μL MACS buffer (protein-free PBS containing 0.2% BSA and 2 mM EDTA) and labeled with 20 μL CD14 MicroBeads for every 1 × 10^7^ cells. The labeled cells were incubated for 15 min at 4 °C and were then washed in 1 mL MACS buffer. The PBMCs were resuspended in 500 μL MACS buffer for every 1 × 10^8^ cells and separated through an MS column as per the manufacturer’s instructions. For quality control of the efficiency of monocyte enrichment, aliquots of whole PBMCs (pre-enrichment) and CD14^+^ monocytes (post-enrichment) were stained with a fluorescently labeled anti-CD14 antibody for flow cytometry analysis (Supplementary Fig. 1b).

### In vitro differentiation of moMΦs

Magnetically enriched CD14^+^ monocytes were plated at a density of 1–1.5 × 10^6^ cells per well in 12-well tissue culture-treated plates (TPP) in RPMI-1640 medium containing 5% human AB serum (Sigma Aldrich), 5% FSC (PAN Biotech), 1% Penicillin/Streptomycin (PAN Biotech), 10 mM HEPES (Gibco) and 20 ng/mL human recombinant M-CSF (BioLegend). This culture medium is referred to as a complete RPMI medium. Monocyte cultures were incubated at 37 °C and 5% CO_2_. Fresh medium was supplemented on days 2, 4, and 6 of differentiation. On day 7, adherent moMΦs were carefully washed in PBS and dissociated from the plates using Cell Dissociation Buffer (Life Technologies Corporation). For cell dissociation, 500 μL of Cell Dissociation Buffer was added per well, and cells were incubated for 15 minutes at room temperature, occasionally tapping the plate to facilitate cell detachment. The cells were then gently dissociated by simultaneous pipetting and mixing of the buffer and scraping of the bottom of each well with the pipette tip. The buffer containing the detached cells was transferred in fresh 2 mL tubes. The wells were washed in 1 mL PBS, adding the PBS was put back into the cell suspension. The cells were then centrifuged at 350×*g* for 10 min. The buffer was carefully removed without disturbing any cell pellet, and the cells were resuspended in 1 mL complete RPMI medium. Dissociated moMΦs were counted, re-plated in 48-well cell culture-treated plates at a density of 5 × 10^5^ cells per well, and incubated at 37 °C for 1 h to allow for re-adherence to the plates prior to cell stimulation and infection.

### Cell stimulation and virus infections

All work with wild-type MARV371 (referred to herein as MARV) and recombinant MARV-ZsG filoviruses was conducted at the Robert Koch Institute under Biosafety Level 4 (BSL-4) laboratory conditions. Research staff involved in this study adhered closely to approved BSL-4 safety protocols and standard operation procedures (SOPs) for sample inactivation and removal from the BSL-4 facility.

For virus infection herein, in vitro-differentiated moMΦs were covered in a minimal volume of complete RPMI culture medium and were exposed to either the wild-type bat isolate MAR371 (isolate Uganda 200704852 Uganda Bat, referred to in this study as MARV) or with a recombinant fluorescent MARV371 expressing ZsGreen^22^(referred to in this study as MARV-ZsG) at a multiplicity of infection (MOI) of 2 (as titrated on Vero E6 cells) for 1 h at 37 °C and 5% CO_2_. After 1 h of exposure, the virus inoculum was removed, the cells were washed in PBS, 250 μL/well of fresh complete RPMI medium was added, and the cells were returned to the incubator. For stimulation with LPS, cultured moMΦs were incubated in a complete RPMI medium containing 2 μg/mL LPS (InvivoGen). For mock-infected controls, moMΦs were incubated in a complete RPMI medium alone. After 24 h of incubation (1 day post-infection), cells and supernatants were harvested and used for further analysis. MARV-ZsG-infected moMΦs were visualized on an EVOS cell imaging system (Thermo Fischer Scientific).

### Flow cytometry

For flow cytometric measurement of cell surface marker expression, mock-infected and MARV-ZsG-infected moMΦs were dissociated from cell culture plates as described above. Cells were then stained in 30 μL/sample of antibody mix in FACS buffer (protein-free PBS containing 0.2% BSA and 2 mM EDTA) with LIVE/DEAD Fixable Yellow Dead Cell Stain Kit (Invitrogen) and anti-human antibodies raised against the following markers: CD11b-AF594 (clone M1/70 diluted 1:100, BioLegend), CD14-PerCP (clone HCD14 diluted 1:100, BioLegend), CD40-PE-Cy7 (clone 5C3 used at 2 μL/sample, BioLegend), CD163-AF647 (clone QA19A16 diluted 1:100, BioLegend), CD206-PB (clone 15-2 diluted 1:100, BioLegend) and HLA-DR-A785 (clone L243 diluted 1:50, BioLegend). Cells were stained for 15 min at room temperature, followed by washing once in 200 μL/sample of FACS buffer and fixed overnight in 200 μL/sample of 10% formalin. Fixed cells were centrifuged and transferred in fresh 200 μL formalin for removal from the BSL-4 laboratory following approved SOPs. Stained samples were run on a Cytoflex S cytometer (Supplementary Fig. 2a, Beckman Coulter GmbH). Flow cytometry data were analyzed using FlowJo software version 10.8.1 (TreeStar).

### sCD163 ELISA

To detect sCD163, 100 μL per well of cell culture supernatants were collected from mock-infected, LPS-treated, and MARV-infected moMΦs and stored at −80 °C. Concentrations of sCD163 in these samples were quantified under BSL-4 laboratory conditions using the Human CD163 Uncoated ELISA kit (Invitrogen) following the manufacturer’s instructions, using a 1:8 sample dilution (Supplementary Fig. 2b).

### Gene expression analysis

For bulk RNA sequencing (RNAseq), mock-infected, LPS-treated, and MARV-infected moMΦs were lysed in 350 μL RLT buffer. For sample inactivation, 600 μL of 70% ethanol was added to the sample-RLT mix, and samples were removed from the BSL-4 facility following approved SOPs. RNA was extracted using the QIAGEN RNeasy Mini Kit according to the manufacturer’s instructions, and samples were submitted to Novogene for library preparation, quality control, and sequencing. Messenger RNAs (mRNA) were purified from total RNA using poly-T oligo-attached magnetic beads. Following fragmentation, the first strands of complementary DNAs (cDNA) were synthesized using random hexamer primers, followed by the second cDNA strand synthesis, end repair, A-tailing, adapter ligation, size selection, amplification, and purification. After final quality control, cDNA libraries were sequenced on multiple lanes using an Illumina NovaSeq platform.

Quality control of the obtained RNAseq reads was performed using FastQC^45^. Trim Galore was used to trim index adaptors and to remove any low-quality base calls or reads below 20 base pairs using a read quality cutoff Phred score of 33^46^. Trimmed, quality-controlled reads were merged into a single file for each sample and aligned against the GRCh38 (hg38) human reference genome. For viral gene counts, trimmed and filtered reads were aligned against the MARV ViralProj15199 reference genome. Gene-level counts were quantified using Kallisto^47^. Gene counts were filtered, and log_2_ normalized using the Tidyverse, BaseR, and EdgeR packages in RStudio^48,49^. Differential gene expression analysis was performed using the Bioconductor package DESEQ2 to identify genes differentially expressed between mock-infected, LPS-treated, and MARV-infected human moMΦs^50^. Volcano plots were generated using the ggplot2 package and illustrate log_2_ fold change differences, where genes with a log_2_ fold change above 2 or below −2 are represented in red and blue, respectively. In heatmaps and volcano plots, only DEGs with an adjusted p-value < 0.05 were included. Functional annotation of biological processes and molecular functions of differentially expressed genes was performed using DAVID^24,25^.

### CIBERSORTx analysis

Normalized log2 gene counts were uploaded for analysis using the web-based Cell-type Identification by Estimating Relative Subsets of RNA Transcripts (CIBERSORTx, https://cibersort.stanford.edu/, Stanford University, Stanford, CA, USA). The analysis was performed using a reference set of 22 immune cell subtypes and was run for 100 permutations. The results graph was prepared using GraphPad Prism (Supplementary Fig. 3, GraphPad Prism Software, Inc., La Jolla, CA, USA).

### Real-time quantitative PCR (qPCR)

For real-time quantitative PCR (qPCR) of viral RNA in cell culture supernatants, 140 μL of cell culture supernatant was collected from mock-infected and MARV-infected moMΦs and was added to 560 μL AVL buffer (Qiagen). For sample inactivation, 560 μL of 100% ethanol was added to the sample–AVL mix, and samples were then removed from the BSL-4 facility following approved SOPs. RNA was extracted using the QIAamp Viral RNA Kit (Qiagen), following the manufacturer’s instructions. MARV transcripts were quantified using a qPCR assay targeting MARV-NP using an AgPath-ID One-Step RT-PCR Kit (Thermo Fischer Scientific). Reactions of 25 μL were formulated by adding 5 μL of sample to a master mix containing 10 μM forward and reverse primers, 10 μM of TaqMan probe, 1× buffer, and 1× RT-PCR enzyme mix. The thermal profile included incubation at 45 °C for 15 min, 95 °C for 1 min, and 45 cycles of 95 °C for 15 s and 60 °C for 60 s. Sample CT values were compared to a standard curve using MARV in vitro transcripts of known concentrations ranging from 10^1^ to 10^6^ copies. Using the standard curve, gene copies per μL cell culture medium were calculated.

### Luminex assay

One day following LPS treatment, MARV infection, or mock infection, cell culture supernatants from moMΦs were collected and stored at −80 °C. For virus inactivation, 50 μL of the sample was diluted in 25 μL of buffer containing Triton X-100 and Tween-20 with a final concentration of 0.5% for each detergent. The diluted samples were heated to 60 °C for 30 min and removed from the BSL-4 laboratory following approved SOPs. Samples were analyzed using the ProcartaPlex™ Human Cytokine & Chemokine Panel 1A 34-plex kit from Thermo Fischer Scientific, following the manufacturer’s instructions. Mean fluorescence intensity was measured on a Bioplex 200 (BioRad), and final concentrations of each analyte were calculated in pg/mL.

### Statistical analysis

Statistical analysis of flow cytometry data was performed using GraphPad Prism software version 9.1.0 (La Jolla, CA, USA). Results in the Box-and-Whiskers plot in Fig. 2c were tested for normality using the Shapiro–Wilk normality test, followed by a Wilcoxon Signed Rank test. The Luminex data in Fig. 6 were analyzed using a Kruskall–Wallis multiple comparisons test. **p < 0.01, ***p < 0.001.

## Supplementary information

Supplementary figures

Supplementary Table1

Supplementary Table2

## Data Availability

All data in this study are available from the corresponding author upon reasonable request.

## Code availability

The underlying code used for RNAseq data analysis in this study is available on GitHub.
