# Supplementary material for: Human macrophages infected with Egyptian Rousette bat-isolated Marburg virus display inter-individual susceptibility and antiviral responsiveness
Source: Npj Viruses. 2024 May 2;2:19. doi: 10.1038/s44298-024-00027-3 (PMC11721647; doi:10.1038/s44298-024-00027-3)
Supplement: Supplementary file 1 — Supplementary figures [file 44298_2024_27_MOESM1_ESM.docx]

**Human macrophages infected with Egyptian rousette bat-isolated Marburg virus display inter-individual susceptibility and antiviral responsiveness**

**Supplementary figures**

Ivet A. Yordanova^1^, Angelika Lander^1^, Annette Wahlbrink^1^, Jonathan S. Towner^2^, César G. Albariño^2^, Lay Teng Ang^3^, Joseph B. Prescott^1*^

^1^ Centre for Biological Threats and Special Pathogens, Robert Koch Institute, 13353 Berlin, Germany

^2^ Viral Special Pathogens Branch, Centers for Disease Control and Prevention, Atlanta, GA 30329, USA

^3^ Stanford Institute for Stem Cell Biology & Regenerative Medicine, Stanford University, Stanford, CA 94305 USA

* Corresponding author


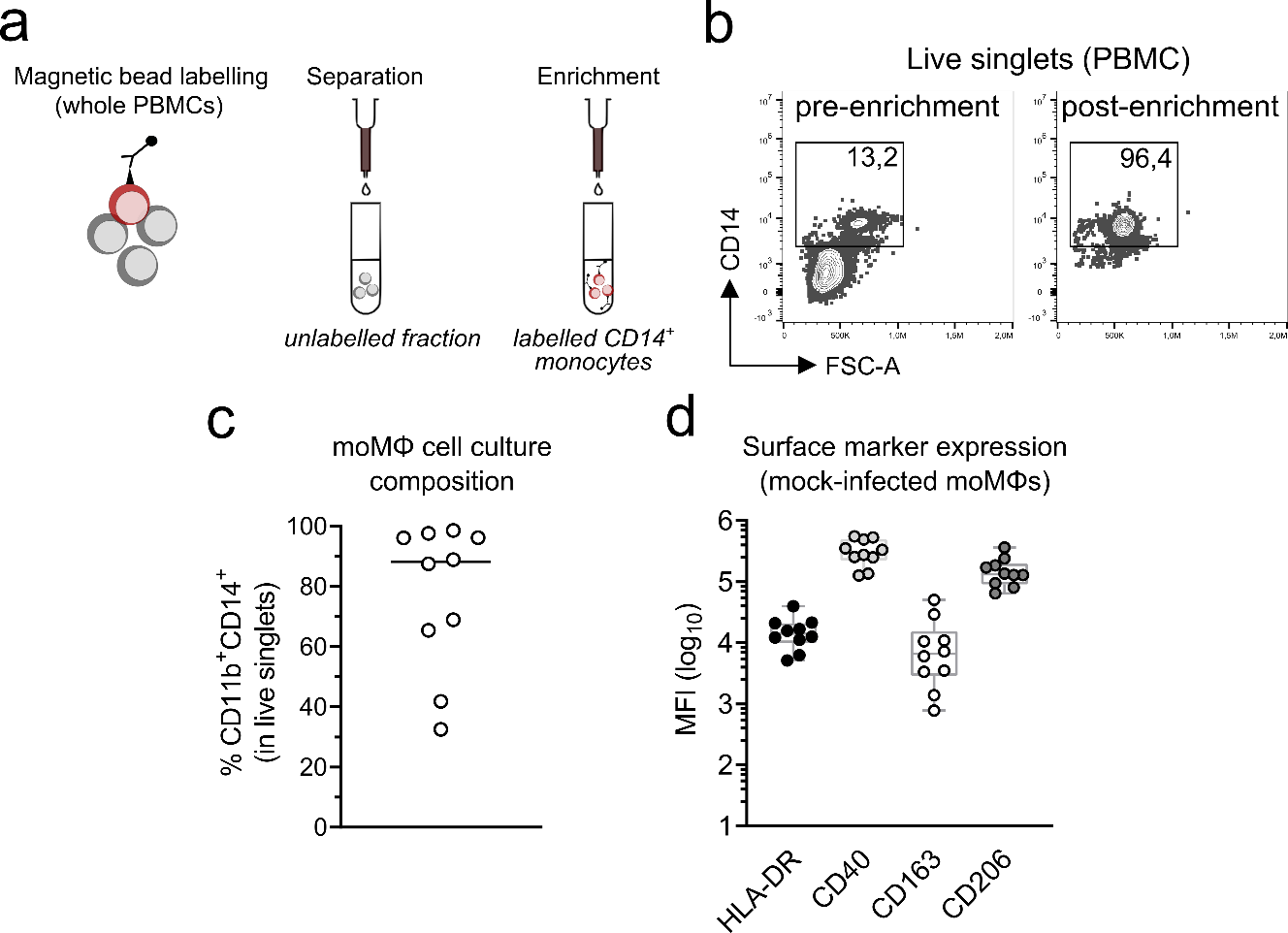


**Supplementary Figure 1.** Human CD14^+^ monocyte enrichment. (**a**) Graphical representation of the workflow for CD14^+^ monocyte magnetic bead enrichment from human PBMCs. Briefly, whole PBMC fractions were collected from fresh blood samples following density gradient separation. PBMCs were washed and labelled with anti-CD14 magnetic beads and were added to MS columns on a Miltenyi OctoMACS separator. Unlabelled fractions were discarded and labelled CD14^+^ monocytes were collected for further differentiation. (**b**) Example FACS plots of the fraction of CD14^+^ monocytes in PBMC samples pre- and post-enrichment. (**c**) Percentage of CD11b^+^CD14^+^ cells in moMΦ cultures from 10 individual blood donors, quantified within live singlets via flow cytometry. (**d**) Median fluorescence intensity (MFI) of HLA-DR, CD40, CD163 and CD206 expressed on the surface of moMΦs from 10 individual donors.


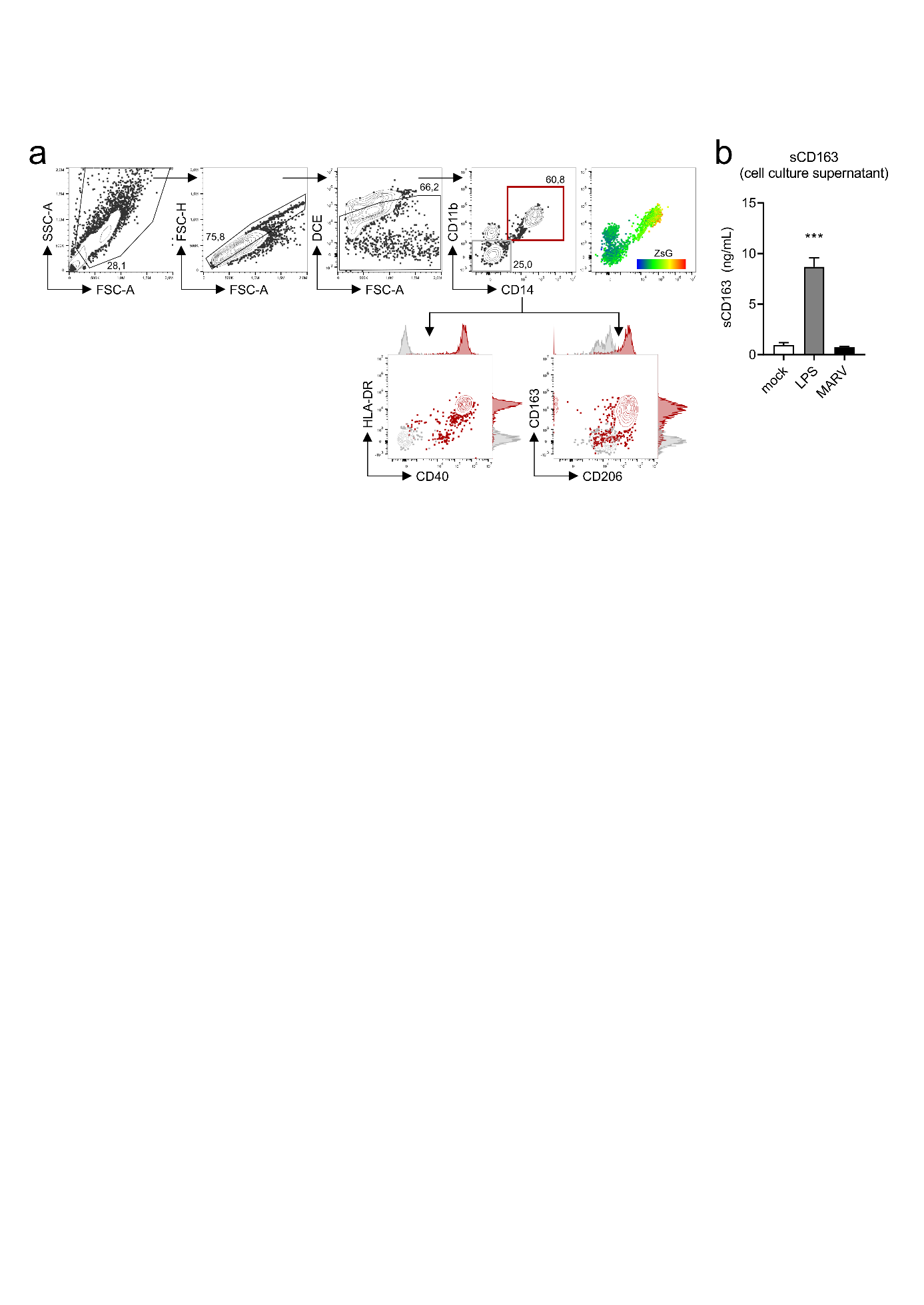


**Supplementary Figure 2.** (a) Gating strategy for the analysis of surface marker expression of moMΦs. Cells were gated based on their forward (FSC-A) and side scatter (SSC-A) for size and granularity. Singlets were gated based on FSC-A and FSC-H. Dead cells were excluded using a Fixable Yellow Viability Dye (dead cell exclusion, DCE). moMΦs were then defined as CD11b^+^CD14^+^ live singlets. Example contour plots of their expression of HLA-DR, CD40, CD163 and CD206 are shown in the color-coded panels below the gating strategy. A color-coded heatmap dotplot illustrates ZsG signal expression within the cell populations in the MARV-infected moMΦ culture. (b) Levels of secreted CD163 (sCD163) in cell culture supernatants of mock-infected, LPS-treated and MARV-infected moMΦs. The data include samples from 3-5 donors per condition. Statistical analysis was performed using a Kruskall-Wallis multiple comparisons test. ***p<0.001.


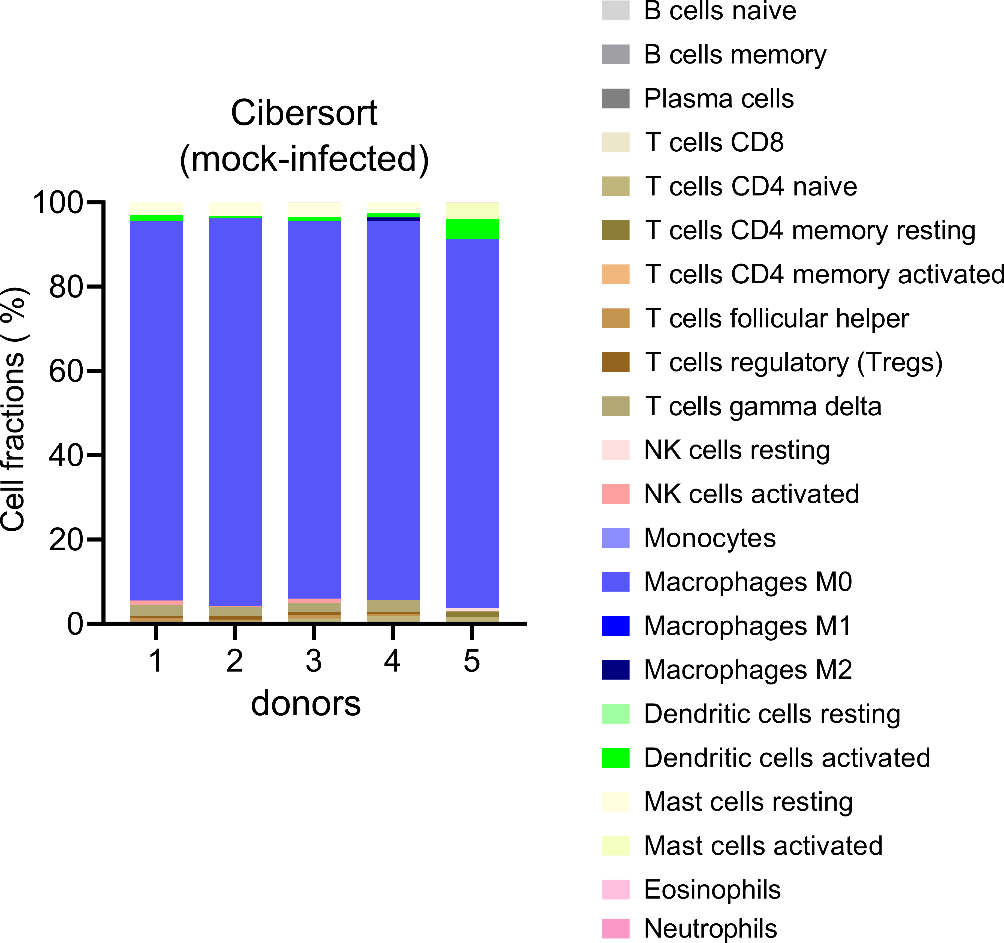


**Supplementary Figure 3.** CIBERSORTx analysis of the baseline cell subset composition of moMΦ cultures using the RNAseq data from the mock-infected samples from the 5 sequenced donors.
